# Robust PLS Prediction Model for Saikosaponin A in *Bupleurum chinense* DC. Coupled with Granularity-Hybrid Calibration Set

**DOI:** 10.1155/2015/583841

**Published:** 2015-03-02

**Authors:** Zhisheng Wu, Min Du, Xinyuan Shi, Bing Xu, Yanjiang Qiao

**Affiliations:** ^1^Beijing University of Chinese Medicine, Beijing 100102, China; ^2^Key Laboratory of TCM-Information Engineering of State Administration of TCM, Beijing 100102, China; ^3^Beijing Key Laboratory for Basic and Development Research on Chinese Medicine, Beijing 100102, China; ^4^World Federation of Chinese Medicine Societies, Beijing 100101, China

## Abstract

This study demonstrated particle size effect on the measurement of saikosaponin A in *Bupleurum chinense* DC. by near infrared reflectance (NIR) spectroscopy. Four types of granularity were prepared including powder samples passed through 40-mesh, 65-mesh, 80-mesh, and 100-mesh sieve. Effects of granularity on NIR spectra were investigated, which showed to be wavelength dependent. NIR intensity was proportional to particle size in the first combination-overtone and combination region. Local partial least squares model was constructed separately for every kind of samples, and data-preprocessing techniques were performed to optimize calibration model. The 65-mesh model exhibited the best prediction ability with root mean of square error of prediction (RMSEP) = 0.492 mg·g^−1^, correlation coefficient (*R*_*P*_) = 0.9221, and relative predictive determinant (RPD) = 2.58. Furthermore, a granularity-hybrid calibration model was developed by incorporating granularity variation. Granularity-hybrid model showed better performance than local model. The model performance with 65-mesh samples was still the most accurate with RMSEP = 0.481 mg·g^−1^, *R*_*P*_ = 0.9279, and RPD = 2.64. All the results presented the guidance for construction of a robust model coupled with granularity-hybrid calibration set.

## 1. Introduction

Near infrared (NIR) reflectance spectroscopy is widely used for quality assessment of solid sample in areas of pharmaceuticals, agriculture, food, fruits, forage, and so on due to its rapid measuring speed, flexibility, and less or even no sample preparation [1–3]. This technology has also shown many applications in Chinese herbal medicine (CHM), including quality control of raw materials [4], manufacturing process control [5–8], and quality assessment of final dosage form [9]. Before NIR analysis, sample preparation of CHM is vital because CHM shape was irregular with coarse surface. Sample preparation was performed by crushing the sample into powder and controlling the particle size by passing the ground powder through sieves so as to keep the consistency of sample presentation.

However, for sample presentation of CHM, different particle sizes affected sample homogeneity, sample packing density, and sample surface, which all introduced uncontrolled variations that brought forth difference in optical path length and multiplicative light scattering effects [10, 11]. Several mathematical methods such as multiplicative scatter correction (MSC) [12], standard normal variate (SNV), extended multiplicative scatter correction (EMSC) [13], orthogonal signal correction (OSC) [14], and optical path length estimation and correction (OPLEC) [15] have been used to mitigate light scattering effects. But the degree of the scattering effects to be mitigated was different according to different granularity effect of sample.

In addition, the fact that sample presentation to the instrument (e.g., particle size) has been found to affect the characteristics of NIR spectra should be paid great attention, thus determining the robustness and accuracy of NIR as analytical technique. According to the effect of soil particle size (SPS) on the NIR measurement of exchangeable sodium (Na), NIR accuracy for soils with great particle sizes (SPS-0.212, 0.212 mm) was higher than soil with small particle sizes (SPS-0.053, 0.053 mm) [16].

Therefore, how to guarantee low noise and good NIR model performance with different granularity effect was worth clarification. Researches concerning this issue have done limited work to give conduction in CHM. David reported a method for quantifying the median particle size of a dry powder using preprocessing NIR spectra. A quadratic model was developed to explain these summations as a function of median particle size, since the effect of densification was minimal [17]. In addition, Sarraguça et al. compared the estimation of the particle size distribution of a pharmaceutical powder using NIR. The estimations were made by considering the former data blocks separately and together using a multiblock approach [18]. Furthermore, particle size determination of amoxicillin trihydrate particles was developed by Bittner. A linear coherence between particle size and absorbance signal was found at specific wavenumbers [19].

Nevertheless, this is only one paper on the particle size of CHM in NIR measurement, which illuminates the influence of granularity on NIR spectra characteristic of* Coptis chinensis* [20]. Few studies focused on the effect of granularity on the quantitative analysis of active pharmaceutical ingredients (API) in CHM, and there was not a globally accepted method that guided the crushing process.

*Bupleurum chinense* DC. is a well-known CHM and is used in at least 66% of the prescriptions in Chinese medicine and Kampo medicine [21]. Saikosaponin was demonstrated to be the major active ingredient in* Bupleurum chinense* DC. Therefore, the content of saikosaponin A (SSA) was quantitatively analyzed by NIR technique with different sized samples with the aim of presenting a methodology to investigate the effects of granularity on different NIR frequency range. Partial least squares (PLS) regression analysis with incorporating samples of various granularities into calibration set was developed for low content of SSA of* Bupleurum chinense* DC.

## 2. Materials and Methods

### 2.1. Sample Preparation

All* Bupleurum chinense* DC. samples were collected from different growing places of China to give increased geographical variations. All the samples were identified by Dr. Chunsheng Liu (Beijing University of Chinese Medicine, China). Sample origins and the numbers of samples are shown in Table 1.

After being cleaned by brushing off soil dust from the surface,* Bupleurum chinense* DC. was crushed into pieces by a disintegrator. Then the samples were ground to fine pieces with a blender and screened through a 20-mesh sieve. Finally, the powders were divided into four parts. Every part was continually smashed and screened through 40-, 65-, 80-, and 100-mesh sieve, respectively.

### 2.2. NIR Spectra Acquisition

About 1 g sample powder was packed into the sample cup. NIR spectra were acquired in reflectance mode with the Integrating-Sphere module of the Antaris I FT-NIR analyzer (Thermo Fisher, USA). Each spectrum was the average of 64 successive scans with air as the background. The spectral range was 10000–4000 cm^−1^ with 1.928 cm^−1^ data interval. To guarantee the analysis accuracy, each sample was analyzed in triplicate and the mean value of three spectra was used in the following analysis. To avoid the effects of environment condition in the laboratory, such as temperature and humidity, the room temperature was controlled at 25°C, and the humidity was kept at an ambient level.

### 2.3. Reference Analysis Method

The reference method used for SSA determination was the high performance liquid chromatography (HPLC) assay recommended by the Chinese Pharmacopoeia (ChP, 2010 Edition) for* Bupleurum chinense* DC. Amounts of SAA (12.5 mg) were accurately weighed using an XS205DU electronic balance (Mettler Toledo, Greifensee, Switzerland) and dissolved with methanol into a 25 mL volumetric flask. Chromatographic analysis was conducted on a Wondasil C18 column (250 mm × 4.6 mm, 5 *μ*m, SHIMADZU, Japan) at 30°C using an Agilent 1100 series HPLC apparatus, equipped with a quaternary solvent delivery system, an autosampler, and a DAD detector. The detection wavelength was 210 nm. With a flow rate of 1.0 mL/min, the linear gradient elution program was set, as shown in Table 2.

### 2.4. Data Pretreatment and Analysis

All the computations were performed using TQ Analyst software package (version 8.0, Thermo Scientific, Madison, USA). Other data analyses were performed by Unscrambler 9.7 software package (Camo Software AS, Norway) and MATLAB version 7.0 (MathWorks Inc., USA). Some of the algorithms used in this paper were developed by us.

## 3. Results and Discussion

### 3.1. Chromatographic Studies on* Bupleurum chinense* DC

**Figure 1:**
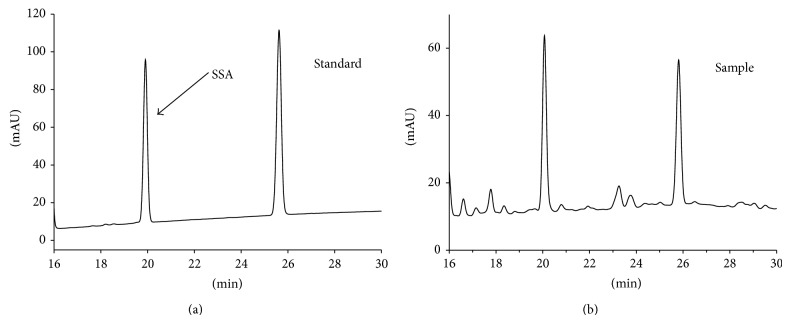
The chromatograms of* Bupleurum chinense* DC. extraction solution.

Figure 1 shows typical HPLC chromatograms of* Bupleurum chinense* DC. extraction solution. The retention time of the SSA in the sample solution was the same with the reference standard solution. The calibration curve of the HPLC method was investigated before real sample analysis. The calibration curve exhibited good linearity (*y* = 0.0031*x* + 0.0126, *R* ^2^ = 0.9999) within the content range 0.804–6.432 *μ*g.

### 3.2. Effects of Granularity on Absorption Characteristics of Overtones and Combination of NIR

**Figure 2:**
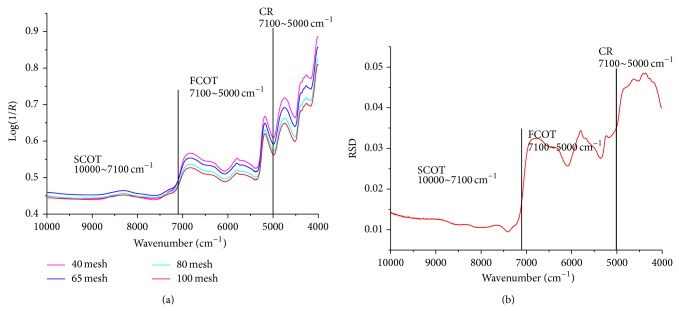
(a) Raw spectra of samples with different granularity. (b) Difference of NIR frequency range to the granularity.

Figure 2(a) shows typical raw spectra of one sample with different granularity.  Figure 2(b) describes overtones and combination characteristics of NIR spectra to the granularity. It was obvious that the difference of spectral characteristics was closely related to granularity. The effects of granularity were wavelength dependent. According to Kubelka-Munk function (1), reflectance was inversely proportional to the light scatter coefficient *S*:

(1) $$\begin{array}{c} Ϝ\left(R_{\infty }\right)=\frac{{\left(1-R_{\infty }\right)}^{2}}{2R_{\infty }}=\frac{K}{S}.\; \end{array}$$

Former research demonstrated that *S* value was inversely proportional to particle size [22]. Therefore, Log(1/*R*) value was proportional to particle size. However, Figure 2 shows that this principle was only effective for NIR spectra of* Bupleurum chinense* DC. in the first combination-overtone region (FCOT, 7100–5000 cm^−1^) and combination region (CR, 5000–4000 cm^−1^).

It could be observed that Log(1/*R*) value was sensitive to granularity changes, which tended to become larger as the particle size increased. Compared with FCOT region (RSD, 0.025–0.035), NIR absorption of CR region was more easily interfered with by granularity (RSD, above 0.035). However, in the second combination-overtone region (SCOT, 7100–10,000 cm^−1^), Log(1/*R*) value was relatively steady and not vulnerable to disturbance (RSD, less than 0.015).

### 3.3. Optimization of NIR Data-Preprocessing Methods

To avoid bias in sample selection, the Kennard-Stone (KS) algorithm was used to split the NIR data set into calibration and validation. Twenty concentration levels including 60 samples were used as the calibration set, and the remaining samples were the validation set, which was shown in Table 3. Outliers were firstly removed before model calibration according to Dixon test. Dixon test is defined as that if the deviation of a standard from the mean is outside a 95% confidence threshold, the standard is an outlier.

**Figure 3:**
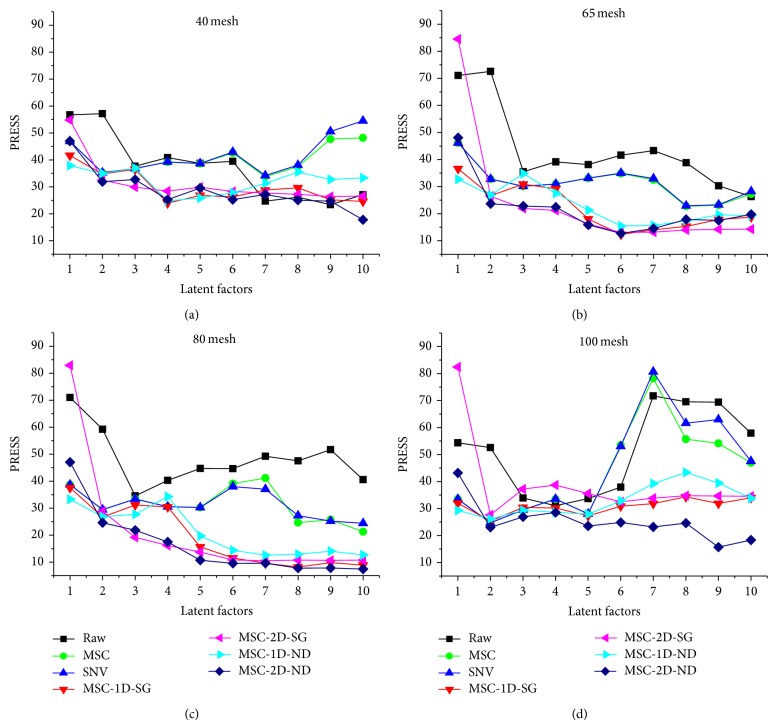
Plot of PRESS value against latent factors.

Data-preprocessing techniques were investigated prior to calibration development. To optimize the spectra, the empirical multiplicative light scattering correction method, MSC, and SNV were applied. Then combination of derivative methods including first derivative (1D) and second derivative (2D) was used to reduce baseline variations observed in original diffuse reflectance spectra and to enhance spectral features. Meanwhile, smoothing methods including Savitzky-Golay smoothing filter (SG) and Norris derivative filter (ND) were employed to depress the background noise amplified by derivative. The optimum preprocessing method was determined by the lowest PRESS value (Figure 3). It was concluded that, for* Bupleurum chinense* DC. of different granularity, the optimization result was a little different.

### 3.4. Effects of Granularity on Local PLS Model Prediction Ability

**Figure 4:**
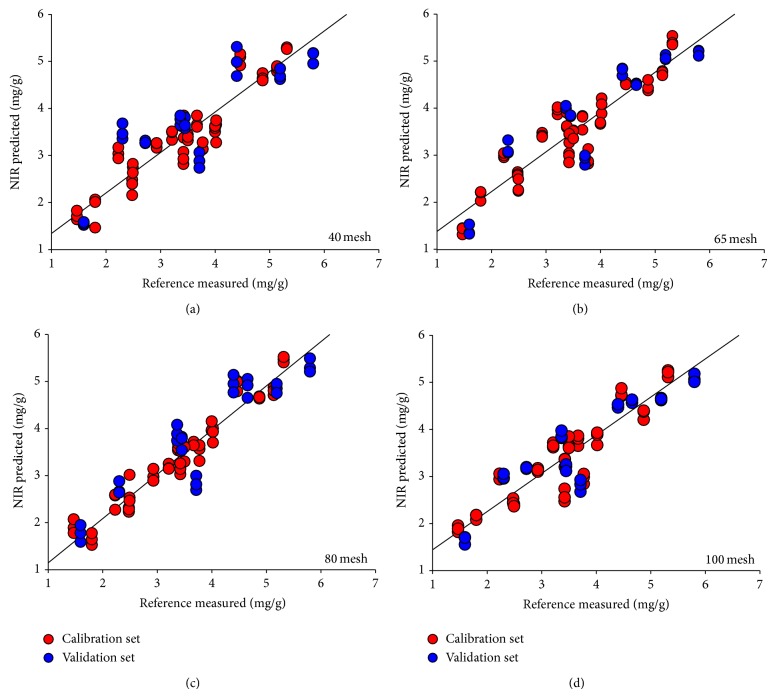
Correlation diagrams between the NIR predicted values and the reference values of SSA content.

After application of the best data pretreatments, four local PLS models were constructed with powder samples, which were screened through 40-, 65-, 80-, and 100-mesh sieve separately. To compare the prediction performance of every local model, test-set validation was performed and the result was shown in Table 4. The correlation diagram was shown in Figure 4. To avoid overfitting phenomenon, RMSECV value was closed to RMSEP when determining the principle component numbers.

It was significantly found that local model performance was not gradually increasing with decreased granularity. The result demonstrated that model performance went down at 65 mesh and tended to be steady from 80 mesh to 100 mesh. The result showed that granularity and sample heterogeneity were both critical for NIR analysis. When grinding the solid sample, sample granularity should be considered. Furthermore, the local model was not very perfect though its correlation coefficient was greater than 0.9. To further improve model performance, granularity-hybrid calibration model was tried in the next section.

### 3.5. Construction of Granularity-Hybrid Calibration Model

To develop a robust calibration model and realize model's successful application, another way to defend variations of particle sizes is to construct a granularity-hybrid calibration model (GH model), including calibration set of every granularity (240 samples, 40, 65, 80, and 100 mesh). Then validation sets of every particle size were predicted by the GH model, as shown in Figure 4. RMSEP and *R*_*p*_ were compared to find whether model with granularity-hybrid sample set could be more accurately predicted.

**Figure 5:**
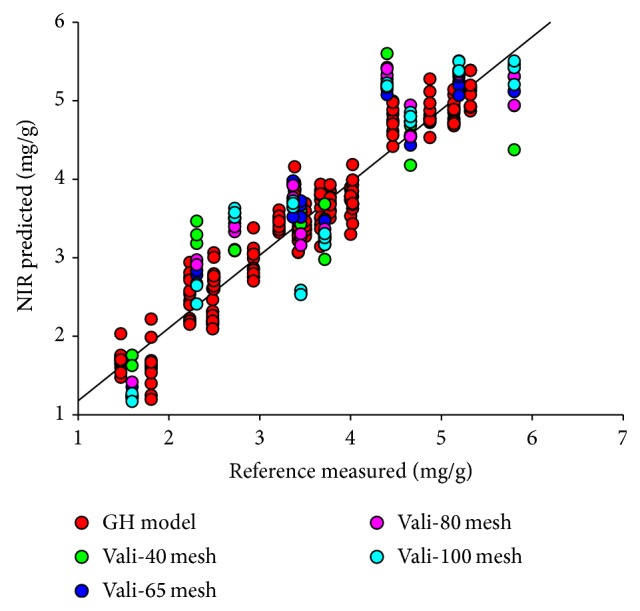
Correlation diagrams of GH model.

GH model performance constructed with different data-preprocessing methods was exhibited in Table 5. We concluded that MSC + 1D + SG was the best method for GH model development. The correlation diagram of GH model was shown in Figure 5. Similarly conclusion has shown that model performance of 65-mesh sample could be the most accurately predicted based on the chemometric indicators. The 80-mesh and 100-mesh samples' prediction results showed no significant difference ranking the second. Furthermore, the prediction performance of 40-mesh samples was still the worst. The above results illuminated basic guidance for sample preparation. It was obvious that GH model was better than local model, which demonstrated that hybrid calibration model was a good alternative to deal with variations.

## 4. Conclusions

Effects of granularity on NIR were investigated; the results concluded that influence on NIR spectra was wavelength dependent. NIR intensity was proportional to particle size in the FCOT and CR region. After appropriate data preprocessing, the local PLS model of 65-mesh samples showed the best prediction ability for* Bupleurum chinense* DC. Furthermore, a granularity-hybrid calibration model was developed by incorporating granularity variation. It showed that model performance of hybrid calibration model was better than local model, which demonstrated that hybrid calibration model was a good alternative to deal with variations. All the results present guidance for sample preparation in NIR analysis of CHM and reference for construction of a robust model eliminating granularity factors.

## Figures and Tables

**Table 1:** A summary of tested samples.

Sample number	Origins	Growth pattern
1~5	Shanxi	Unknown
6~9	Shanxi	Unknown
10~14	Shanxi	Cultivated
15~19	Shanxi	Wild
20~25	Shanxi	Wild
25~30	Hebei	Cultivated

**Table 2:** Elution gradient used in the HPLC method.

Time/min	ACN (v/v)	Water (v/v)
0–50	25–90	75–10
50–55	90	10
55–60	25	75
60–67	25	75

**Table 3:** Concentration range of SSA in calibration and validation set (mg·g^−1^).

Sample set	Numbers	Concentration range	Average	Standard deviation
Calibration	60	1.476–8.162	3.695	1.452
Validation	30	1.601–5.807	3.727	1.269

**Table 4:** Local model performance of different granularity.

Model	LVs	Cross validation		Test-set validation		RPD
		RMSECV	*R*_CV_	RMSEP	*R*_*P*_	
40	4	0.682	0.7671	0.650	0.8519	1.95
65	4	0.574	0.8347	0.492	0.9221	2.58
80	3	0.567	0.8408	0.534	0.9070	2.38
100	3	0.664	0.7484	0.522	0.9162	2.43

**Table 5:** Prediction performance of GH model.

Pretreatment methods	LVs	Cross validation		Test-set validation (40)		Test-set validation (65)		Test-set validation (80)		Test-set validation (100)	
		RMSECV	*R*_CV_	RMSEP	*R*_*P*_	RMSEP	*R*_*P*_	RMSEP	*R*_*P*_	RMSEP	*R*_*P*_
RAW	5	0.763	0.6903	0.814	0.7657	0.747	0.8031	0.707	0.8292	0.635	0.8862
MSC	2	0.716	0.7278	0.716	0.8293	0.654	0.8551	0.621	0.8628	0.538	0.9123
1D + SG	7	0.602	0.8229	0.687	0.8420	0.621	0.8473	0.566	0.8909	0.566	0.8929
2D + SG	4	0.624	0.8025	0.671	0.8621	0.596	0.8815	0.612	0.8651	0.540	0.9065
MSC + 1D + SG	6	0.606	0.8276	0.575	0.8971	0.481	0.9279	0.524	0.9137	0.545	0.9146
MSC + 2D + SG	3	0.678	0.7621	0.690	0.8538	0.664	0.8552	0.618	0.8648	0.522	0.9126
MSC + 1D + ND	6	0.609	0.8821	0.672	0.8508	0.580	0.8873	0.631	0.8655	0.765	0.8371
MSC + 2D + ND	6	0.566	0.8450	0.621	0.8757	0.527	0.9099	0.583	0.8863	0.603	0.8879

## References

[1] Stark E., Luchte K., Margoshes M. (1986). Near-infrared analysis (NIRA): a technology for quantitative and qualitative analysis. *Applied Spectroscopy Reviews*.

[2] Roggo Y., Chalus P., Maurer L., Lema-Martinez C., Edmond A., Jent N. (2007). A review of near infrared spectroscopy and chemometrics in pharmaceutical technologies. *Journal of Pharmaceutical and Biomedical Analysis*.

[3] Magwaza L. S., Opara U. L., Nieuwoudt H., Cronje P. J. R., Saeys W., Nicolaï B. (2012). NIR spectroscopy applications for internal and exte rnal quality analysis of citrus fruit-a review. *Food and Bioprocess Technology*.

[4] Li W., Xing L., Cai Y., Qu H. (2011). Classification and quantification analysis of Radix scutellariae from different origins with near infrared diffuse reflection spectroscopy. *Vibrational Spectroscopy*.

[5] Wu Z. S., Xu B., Du M., Sui C., Shi X., Qiao Y. J. (2012). Validation of a NIR quantification method for the determination of chlorogenic acid in *Lonicera japonica* solution in ethanol precipitation process. *Journal of Pharmaceutical and Biomedical Analysis*.

[6] Wu Z., Sui C., Xu B. (2013). Multivariate detection limits of on-line NIR model for extraction process of chlorogenic acid from Lonicera japonica. *Journal of Pharmaceutical and Biomedical Analysis*.

[7] Wu Z. S., Tao O., Dai X., Du M., Shi X. Y., Qiao Y. J. (2012). Monitoring of a pharmaceutical blending process using near infrared chemical imaging. *Vibrational Spectroscopy*.

[8] Laasonen M. (2003). *Near Infrared Spectroscopy, A Quality Control Tool for the Different Steps in the Manufacture of Herbal Medicinal Products*.

[9] Wu Z. S., Du M., Sui C. L. Development and validation of a portable AOTF-NIR measurement method for the determination of Baicalin in Yinhuang oral solution.

[10] Blanco M., Peguero A. (2010). Influence of physical factors on the accuracy of calibration models for NIR spectroscopy. *Journal of Pharmaceutical and Biomedical Analysis*.

[11] Leger M. N. (2010). Alleviating the effects of light scattering in multivariate calibration of near-infrared spectra by path length distribution correction. *Applied Spectroscopy*.

[12] Helland I. S., Naes T., Isaksson T. (1995). Related versions of the multiplicative scatter correction method for preprocessing spectroscopic data. *Chemometrics and Intelligent Laboratory Systems*.

[13] Martens H., Nielsen J. P., Engelsen S. B. (2003). Light scattering and light absorbance separated by extended multiplicative signal correction. Application to near-infrared transmission analysis of powder mixtures. *Analytical Chemistry*.

[14] Wold S., Antti H., Lindgren F., Öhman J. (1998). Orthogonal signal correction of near-infrared spectra. *Chemometrics and Intelligent Laboratory Systems*.

[15] Jin J.-W., Chen Z.-P., Li L.-M. (2012). Quantitative spectroscopic analysis of heterogeneous mixtures: The correction of multiplicative effects caused by variations in physical properties of samples. *Analytical Chemistry*.

[16] Cozzolino D., Morón A. (2010). Influence of soil particle size on the measurement of sodium by near-infrared reflectance spectroscopy. *Communications in Soil Science and Plant Analysis*.

[17] Ely D. R., Thommes M., Carvajal M. T. (2008). Analysis of the effects of particle size and densification on NIR spectra. *Colloids and Surfaces A: Physicochemical and Engineering Aspects*.

[18] Sarraguça M. C., Cruz A. V., Amaral H. R., Costa P. C., Lopes J. A. (2011). Comparison of different chemometric and analytical methods for the prediction of particle size distribution in pharmaceutical powders. *Analytical and Bioanalytical Chemistry*.

[19] Bittner L. K. H., Heigl N., Petter C. H. (2011). Near-infrared reflection spectroscopy (NIRS) as a successful tool for simultaneous identification and particle size determination of amoxicillin trihydrate. *Journal of Pharmaceutical and Biomedical Analysis*.

[20] Hao G., Ma Q., Zhang Z. (2006). Influence on the NIR spectrum of coptis chinensis by different granularity. *Modern Instruments*.

[21] Zhu Z., Liang Z., Han R., Dong J. (2009). Growth and saikosaponin production of the medicinal herb *Bupleurum chinense DC*. under different levels of nitrogen and phosphorus. *Industrial Crops and Products*.

[22] Otsuka M. (2004). Comparative particle size determination of phenacetin bulk powder by using Kubelka-Munk theory and principal component regression analysis based on near-infrared spectroscopy. *Powder Technology*.

